# Prevalence of ideal cardiovascular health and its relationship with relative handgrip strength in rural northeast China

**DOI:** 10.3389/fcvm.2023.1124757

**Published:** 2023-06-02

**Authors:** Jingan Shao, Bin Yao, Zhecong Yu, Jiahui Xu, Jing Wu, Yanan Ma, Liqiang Zheng, Zhaoqing Sun

**Affiliations:** ^1^ Department of Cardiology, Shengjing Hospital of China Medical University, Shenyang, China; ^2^ School of Public Health, China Medical University, Shenyang, China; ^3^ Department of Orthopedics, Shidong Hospital, Shanghai, China; ^4^ Institute for Prevention and Control of Non-Communicable Chronic Diseases, Hangzhou Center for Disease Control and Prevention, Hangzhou, China; ^5^ Ministry of Education-Shanghai Key Laboratory of Children’s Environmental Health, School of Public Health, Shanghai Jiao Tong University School of Medicine, Shanghai, China; ^6^ School of Nursing, Shanghai University of Traditional Chinese Medicine, Shanghai, China; ^7^ Department of Biostatistics and Epidemiology, School of Public Health, China Medical University, Shenyang, China; ^8^ Institute of Health Sciences, China Medical University, Shenyang, China

**Keywords:** handgrip strength, ideal cardiovascular health, cross-sectional study, rural China, follow-up study

## Abstract

**Objectives:**

We aimed to investigate ideal cardiovascular health (CVH), its relationship with handgrip strength, and its components in rural China.

**Methods:**

We conducted a cross-sectional study of 3,203 rural Chinese individuals aged ≥35 years in Liaoning Province, China. Of these, 2,088 participants completed the follow-up survey. Handgrip strength was estimated using a handheld dynamometer and was normalized to body mass. Ideal CVH was assessed using seven health indicators (smoking, body mass index, physical activity, diet, cholesterol, blood pressure, and glucose). Binary logistic regression analyses were performed to assess the correlation between handgrip strength and ideal CVH.

**Results:**

Women had a higher rate of ideal cardiovascular health (CVH) than men (15.7% vs. 6.8%, *P *< 0.001). Higher handgrip strength correlated with a higher proportion of ideal CVH (*P* for trend <0.001). After adjusting for confounding factors, the odds ratios (95% confidence interval) of ideal CVH across increasing handgrip strength tripartite were 1.00 (reference), 2.368 (1.773, 3.164), and 3.642 (2.605, 5.093) in the cross-sectional study and 1.00 (reference), 2.088 (1.074, 4.060), and 3.804 (1.829, 7.913) in the follow-up study (all *P* < 0.05).

**Conclusion:**

In rural China, the ideal CVH rate was low, and positively correlated with handgrip strength. Grip strength can be a rough predictor of ideal CVH and can be used to provide guidelines for improving CVH in rural China.

## 1. Introduction

In 2019, there were an estimated 523 million cases of cardiovascular disease (CVD) and 18.6 million CVD-related deaths globally; thus, CVD remains an important cause of health problems worldwide (1). It has been the major cause of death in China, and the proportion of deaths caused by CVDs continues to increase, particularly in rural populations (2). Studies on CVD risk in rural China demonstrate an alarmingly high prevalence of hypertension, dyslipidemia, and metabolic syndrome. These are relatively high in rural northeast China (3–5). Overall, previous results have shown that 36.2% of adults in rural northeast China have hypertension, 69.4% have at least one type of dyslipidemia, and 34.7% have metabolic syndrome (3–5). Early prevention and intervention are important, because adults who engage in muscle-strengthening activities at various levels show a greatly reduced risk of all-cause and CVD-related mortality. The American Heart Association (AHA) has developed the concept of an ideal cardiovascular health (CVH) score in response to the growing burden of cardiovascular disease (CVD). The goal of the CVH score is to shift attention from reducing the incidence of CVD to improving the overall CVH status of the population (6). CVH can be evaluated using seven health indicators, i.e., nonsmoking status, body mass index (BMI) < 25 kg/m^2^, reaching target levels of physical activity, following a recommended healthy diet, blood pressure <120/<80 mmHg, total cholesterol <200 mg/dl, and fasting blood glucose <100 mg/dl (6). One study showed that the prevalence of individuals with 6–7 ideal CVH metrics in US studies ranged from 0.5% to 12% (7). Studies have shown that the proportion of Chinese adults with ideal CVH (meeting 7 ideal metrics) is very low, and the estimated percentage of ideal CVH is 0.2% (0.1% for males and 0.4% for females) (8). Among the 7 ideal CVH metrics, an ideal diet is the least commonly achieved (8). Another study on an urban Chinese population showed that only 0.5% of participants met the ideal level of all 7 CVH metrics, and 26.9% of participants met 5 to 7 ideal CVH metrics, among which fasting glucose was the most common metric (71.2%) and physical activity was the least common metric (18.1%) (9). Research on industrial cities in northern China shown that 9.1% of the participants achieved 5–7 ideal metrics, while only 0.1% of the participants achieved the 7 ideal CVH metrics (10). A study of Peruvian adults over the age of 35 years showed that none of the 3,058 participants met all 7 ideal CVH metrics, while 10.5% met less than one ideal CVH metric, in which fasting glucose was the most common CVH metric (72%) (11). Poor cardiovascular health (CVH) status can have negative impacts on people's lives. Studies have shown that having an ideal CVH is associated with better prognosis of cardiovascular-related diseases. A study in a Japanese population found that people with ideal CVH had a significantly reduced risk of developing atrial fibrillation (12). Another study found that women who had an increase in relative grip strength had a lower 10-year risk of developing cardiovascular disease (13). Higher relative grip strength is associated with better CVD biomarkers (14), including triglycerides and glucose, in both men and women (14–16) Reduced grip strength correlated with increased all-cause mortality and cardiovascular mortality (17, 18). Therefore, improving CVH status is an urgent public health problem.

Grip strength is associated with ideal CVH metrics. One study showed that adopting a healthy lifestyle (adequate levels of physical activity, regular consumption of fruits and vegetables, drinking less alcohol, and not smoking) can lead to increased muscle strength in adults and older adults (19). Findings from a prospective association study on muscle strength and physical activity, found that grip strength was positively correlated with physical activity at follow-up. The study also found that poor grip strength could independently predict lower activity level at follow-up (20). Studies have shown that grip strength correlated positively with high-density lipoprotein cholesterol (HDL-C) levels (15). Higher relative muscle strength was significantly associated with more favorable CVD biomarkers, including systolic blood pressure, triglyceride, HDL-C, glucose, and plasma insulin levels in both men and women (14). A study assessing muscle strength by grip strength found that absolute muscle strength and highly standardized muscle strength were directly related to diastolic blood pressure (16). Another study on community-dwelling older adults showed that isometric grip strength training resulted in a significant decrease of 9 mmHg in resting systolic blood pressure (21). Studies on older community-dwelling individuals showed that muscle mass and grip strength are significantly negatively correlated with elevated hypertension (22). A different cross-sectional study found that having a higher relative grip strength was associated with lower risks of impaired fasting glucose, elevated triglycerides, abdominal obesity, and general obesity (23).

To the best of our knowledge, only a few studies have analyzed the association between ideal CVH and muscle strength in children, adolescents, and Colombian college students, and the results showed a positive correlation (24, 25). Studies examining the association between handgrip strength and ideal CVH have primarily been conducted in developed countries. However, given the distinct differences in CVH and grip strength levels between China and developed nations, further research is warranted to elucidate the relationship between these factors in the Chinese population. In addition, the prevalence of ideal CVH has not yet been examined among rural Chinese residents. Moreover, previous studies were mostly cross-sectional studies, rather than a follow-up cohort study. The investigation of ideal CVH in rural China can aid local governments in developing relevant prevention strategies. Analyzing the association between relative grip strength and ideal CVH can provide a basis for early intervention to promote strength preservation as part of the original prevention.

Therefore, in this study, we aimed to investigate the prevalence of ideal CVH, as well as the association between handgrip strength and ideal CVH and its components, among rural residents in China in a cross-sectional and follow-up study.

## 2. Materials and methods

### 2.1. Study population and design

This study was based on a cross-sectional and follow-up survey performed in rural areas in Fuxin County, Liaoning Province, China. According to geographical regions, four towns and 33 villages were randomly selected for data collection in 2019 from the east, south, and north regions. Of the four towns selected, two towns were located in the east, one in the south, and one in the north. We included local residents who had lived in the area for more than 5 years; were willing to sign a consent form; and were aged ≥35 years. Pregnant women, individuals with malignant tumors, those with severe hepatic and renal insufficiency, and those unwilling to participate in the study were excluded. Finally, data from 4,689 individuals were collected at baseline. Of the participants included at baseline, 1,309 lacked information on grip strength and were excluded. A total of 177 participants were excluded because data on ideal CVH metrics, such as total cholesterol (*n* = 17), blood pressure (*n* = 8), physical activity (*n* = 145), BMI (*n* = 2), smoking (*n* = 4), and diet (*n* = 1), were missing. At baseline, 3,203 study participants were selected. A follow-up survey was conducted in 2021.

However, 1,844 participants did not participate in the follow-up study. Of these, 146 lacked information on grip strength and were excluded. A total of 452 participants were excluded as data on ideal CVH metrics, such as total cholesterol (*n* = 50), blood pressure (*n* = 311), physical activity (*n* = 87), BMI (*n* = 3), and smoking (*n* = 1), were missing for 2021. Ultimately, 761 participants were included in the final analysis (Supplementary Figure S1).

The procedures of the study were in accordance with the ethical standards of the Committee for Human Experimentation of China Medical University [2018083], and written informed consent was obtained from all participants.

### 2.2. Assessment of ideal CVH

Ideal CVH as defined by the AHA includes four behavioral metrics (smoking, physical activity, BMI, diet) and three biological metrics (total cholesterol, blood pressure, and blood glucose). Self-reported questionnaires were used to collect information on smoking, physical activity, and diet. In this study, we adopted the AHA definition and made several adjustments. In terms of smoking, participants were classified as never smokers (never smoked or quit ≥12 months ago), former smokers (quit <12 months ago), and current smokers. Diet was measured using five components: (1) ≥250 g cereals and potatoes; (2) ≥500 g fruits and vegetables; (3) <75 g meat and poultry/aquatic products; (4) <50 g sugar; (5) <6 g salt; and (6) in accordance with the current “Dietary Guidelines for Chinese Residents” (26). The researchers judged whether the diet was ideal according to the ideal status of the five dietary components, in which achieving 0‒1 dietary components was poor, 2‒3 was intermediate, and 4‒5 was ideal. BMI, measured by a trained medical professional, was calculated as weight (kg) divided by height squared (m)^2^. According to the AHA protocol, after participants had rested for 5 min, a trained and certified observer took blood pressure measurements three times in a sitting position, at measurement intervals of at least 1 min. The average blood pressure value was used in the final analysis. Blood pressure was estimated using a standardized electronic sphygmomanometer (HEM-8102A; Omron, Dalian, China). The researchers obtained fasting blood samples in the morning from participants who had fasted for at least 8 h. Specific criteria are listed in Supplementary Table S1.

### 2.3. Assessment of handgrip strength

The research staff used a handheld dynamometer (Jamar Plus+, Patterson Medical, Warrenville, IL, USA) to measure handgrip strength according to a standardized protocol (27). During the test, participants were asked to sit while the dynamometer was suspended from their necks with their forearms at 90°. The measurement required rapid exertion, and the participants were asked to hold the dynamometer with maximum force and press it for 3 s. Measurements were taken on each hand, three times, at intervals of 30 s. The final results were recorded at the end of the test. The average grip strength from both hands was used for analysis with the handgrip strength normalized to body mass (28, 29).

### 2.4. Statistical analysis

All analyses were performed using IBM SPSS Statistics v26 (IBM SPSS Inc., Chicago, IL, USA). *P *< 0.05 was considered statistically significant. Data are expressed as mean ± standard deviation (SD), frequency, and percentage. When normality and homogeneity assumptions were satisfied, a two-sample *t*-test was used to examine the differences in numerical variables between male and female participants. Continuous variables were compared using one-way analysis of variance, and categorical variables were tested using the chi-squared test. In addition, binary logistic regression was performed to determine the correlation between relative grip strength and ideal CVH after adjusting for factors such as age, sex, education, ethnicity, history of stroke, and history of coronary heart disease. Adjusted odds ratios (ORs) and 95% confidence intervals (CIs) were calculated. At baseline, based on relative grip strength, participants were divided into tripartite groups (group 1: ≤ 0.36, group 2: 0.36–0.46, group 3: > 0.46), with the first group as the reference category. In the follow-up study, based on relative grip strength, participants were divided into tripartite groups (group 1: ≤ 0.37, group 2: 0.37–0.46, group 3: > 0.46), with the first group acting as the reference category. Logistic regression analysis was performed using three models: model 1 was unadjusted, model 2 was adjusted for sex and age, and model 3 was adjusted for sex, age, education, ethnicity, history of stroke, and history of coronary heart disease.

### 2.5. Patient and public involvement

All data were obtained from a cross-sectional and follow-up study conducted in rural areas in Fuxin County, Liaoning Province, China. None of the patients or the public were involved in the design or planning of this study.

## 3. Results

Of the 3,203 participants in the cross-sectional study, 63.2% were women, and the average age was 57.0 ± 9.9 years. Table 1 compares the differences in the characteristics of CVH according to sex. Women participants in this study were younger, less educated, and had lower grip strength than men. However, in the crude analysis, men were more often at the ideal level for all CVH indicators, except smoking and blood pressure. Women were twice as likely as men to have ideal CVH (15.7% vs. 6.8%, *P *< 0.001) (Table 1).

**Table 1:** Characteristics of rural adults in China in the cross-sectional study (mean ± standard deviation or frequency) (*n* = 3,203).

	Male	Female	*P* value
Age, years	59.9 ± 9.8	57.0 ± 9.9	<0.001
Education level, *n* (%)			<0.001
≤primary school	360 (30.6)	921 (45.6)	
>primary school	818 (69.4)	1,100 (54.4)	
Ethnicity, *n* (%)			0.354
Han	765 (65.0)	1,275 (63.1)	
Mongolian	370 (31.4)	656 (32.5)	
Others	42 (3.6)	90 (4.5)	
Body mass, kg	68.1 ± 12.2	61.8 ± 10.5	<0.001
Body mass index, kg/m^2^	24.4 ± 3.7	25.0 ± 4.8	<0.001
Handgrip strength, kg	34.3 ± 8.2	22.2 ± 5.4	<0.001
Handgrip strength/body mass	0.5 ± 0.9	0.4 ± 0.1	<0.001
**Ideal health metrics**			
Smoking	528 (44.8)	1,721 (85.0)	<0.001
Physical activity	194 (16.5)	397 (19.6)	0.086
Body mass index	686 (58.2)	1,030 (50.9)	<0.001
Diet	225 (19.1)	366 (18.1)	0.114
Blood pressure	188 (15.9)	628 (31.0)	<0.001
Fasting plasma glucose	665 (56.4)	1,127 (55.7)	0.191
Total cholesterol	661 (56.1)	1,006 (49.7)	<0.001
Global CVH, *n* (%)			<0.001
0–2 Metrics	542 (46.0)	696 (34.4)	
3–4 Metrics	556 (47.2)	1,010 (49.9)	
5–7 Metrics	80 (6.8)	318(15.7)	

Characteristics of Chinese rural adults are expressed as mean ± standard deviation in continuous variables, and frequencies and proportions in categorical variables. CVH, cardiovascular health.

Tables 2, 3 compare the differences in CVH characteristics by grip strength. Compared with participants with low relative grip strength, those with higher relative grip strength had better BMI, fasting plasma glucose, total cholesterol, and ideal CVH in the cross-sectional as well as in the follow-up study (Tables 2, 3).

**Table 2:** Characteristics of Chinese rural adults in the cross-sectional study (mean ± standard deviation or frequency) (*n* = 3,203).

		Relative handgrip strength in 2019			*P* value
	Total	0.36 ≥ NGS > 0	0.46 ≥ NGS > 0.36	NGS > 0.46	
Age, years	58.1 ± 10.0	60.7 ± 9.6	57.6 ± 9.9	55.9 ± 9.8	<0.001
Education level, *n* (%)					<0.001
≤primary school	1,281 (40.0)	562 (52.7)	426 (40.0)	293 (27.5)	
>primary school	1,918 (59.9)	505 (47.3)	639 (60.0)	774 (72.5)	
Ethnicity, *n* (%)					0.012
Han	2,040 (63.7)	661 (62.0)	663 (62.3)	716 (67.0)	
Mongolian	1,026 (32.0)	370 (34.7)	346 (32.5)	310 (29.0)	
Others	132 (4.1)	35 (3.3)	55 (5.2)	42 (3.9)	
SBP, mm Hg	134.0 ± 18.5	134.0 ± 18.5	130.9 ± 19.0	131.1 ± 17.9	<0.001
DBP, mm Hg		80.2 ± 10.2	79.6 ± 10.7	80.5 ± 10.7	0.090
Fasting plasma glucose, mg/dl	106.3 ± 33.3	110.7 ± 37.9	105.6 ± 32.0	102.6 ± 28.8	<0.001
Total cholesterol, mg/dl	198.4 ± 37.5	203.4 ± 37.8	198.9 ± 38.0	193.1 ± 35.8	<0.001
Body mass index, kg/m^2^	24.8 ± 4.4	26.5 ± 3.7	24.8 ± 3.4	23.2 ± 5.4	<0.001
**Ideal health metrics**					
Smoking	2,249 (70.2)	891 (83.4)	816 (76.5)	542 (50.7)	<0.001
Physical activity	591 (18.5)	221 (20.7)	191 (17.9)	179 (16.8)	0.056
Body mass index	1,716 (53.6)	375 (35.1)	571 (53.5)	770 (72.1)	<0.001
Diet	591 (18.5)	186 (17.4)	194 (18.2)	211 (19.8)	0.364
Blood pressure	816 (25.5)	233 (21.8)	297 (27.8)	286 (26.8)	0.003
Fasting plasma glucose	1,792 (55.9)	499 (46.7)	627 (58.8)	666 (62.4)	<0.001
Total cholesterol	1,667 (52.0)	475 (44.5)	565 (53.0)	627 (58.7)	<0.001
Global CVH, *n* (%)					<0.001
0–2 Metrics	1,238 (38.7)	486 (45.5)	380 (35.6)	372 (34.9)	
3–4 Metrics	1,566 (48.9)	500 (46.8)	523 (49.0)	543 (50.9)	
5–7 Metrics	398(12.4)	82(7.7)	164(15.4)	152(14.2)	

Characteristics of Chinese rural adults are expressed as mean ± standard deviation in continuous variables, and frequencies and proportions in categorical variables.

NGS, normalized handgrip strength; SBP, systolic blood pressure; DBP, diastolic blood pressure; CVH, cardiovascular health.

**Table 3:** Baseline characteristics of Chinese rural adults in the follow-up study (mean ± standard deviation or frequency) (*n* = 761).

		Relative handgrip strength in 2021			*P* value
	Total	0.36 ≥ NGS > 0	0.45 ≥ NGS > 0.36	NGS > 0.45	
Age, years	58.9 ± 9.4	60.9 ± 9.2	58.0 ± 8.8	57.8 ± 9.7	<0.001
Education level, *n* (%)					<0.001
≤primary school	277 (36.4)	122 (48.0)	88 (34.6)	67 (26.5)	
>primary school	484 (63.6)	132 (52.0)	166 (65.4)	186 (73.5)	
Ethnicity, *n* (%)					0.204
Han	495 (65.1)	159 (62.8)	162 (63.8)	174 (68.8)	
Mongolian	246 (32.4)	91 (36.0)	83 (32.7)	72 (28.5)	
Others	19 (2.5)	3 (1.2)	9 (3.5)	7 (2.8)	
SBP, mm Hg	132.4 ± 18.3	134.3 ± 18.8	130.8 ± 18.4	132.0 ± 17.6	0.093
DBP, mm Hg	79.7 ± 10.6	80.2 ± 10.8	79.3 ± 10.4	79.8 ± 10.6	0.625
Fasting plasma glucose, mg/dl	107.1 ± 31.6	112.8 ± 41.2	106.1 ± 27.7	102.5 ± 22.0	0.001
Total cholesterol, mg/dl	199.9 ± 37.3	204.1 ± 37.3	202.6 ± 39.7	193.1 ± 33.7	0.001
Body mass index, kg/m^2^	24.9 ± 3.6	26.7 ± 3.5	24.9 ± 3.1	23.3 ± 3.2	<0.001
**Ideal health metrics**					
Smoking	556 (73.1)	214 (84.3)	194 (76.4)	148 (58.5)	<0.001
Physical activity	144 (18.9)	55 (21.7)	50 (19.7)	39 (15.4)	0.186
Body mass index	390 (51.2)	81 (31.9)	134 (52.8)	175 (69.2)	<0.001
Diet	138 (18.1)	43 (16.9)	47 (18.5)	48 (19.0)	0.882
Blood pressure	193 (25.4)	58 (22.8)	71 (28.0)	64 (25.3)	0.415
Fasting plasma glucose	392 (51.5)	111 (43.7)	134 (52.8)	147 (58.1)	0.005
Total cholesterol	380 (49.9)	109 (42.9)	123 (48.4)	148 (58.5)	0.002
Global CVH, *n* (%)					0.006
0–2 Metrics	313 (41.1)	123 (48.4)	101 (39.8)	89 (35.2)	
3–4 Metrics	365 (48.0)	114 (44.9)	117 (46.1)	134 (53.0)	
5–7 Metrics	83(10.9)	17(6.7)	36(14.2)	30(11.9)	

Characteristics of Chinese rural adults are expressed as mean ± standard deviation in continuous variables, and frequencies and proportions in categorical variables.

NGS, normalized handgrip strength; SBP, systolic blood pressure; DBP, diastolic blood pressure; CVH, cardiovascular health.

### 3.1. Association between grip strength and ideal CVH in the cross-sectional study

Table 4 present the adjusted relationships between grip strength and the Ideal Global CVH Score in the cross-sectional study. As presented in Table 4, the maximum grip strength group had better global CVH [odds ratio (OR), 95% CI: 3.613, 2.580‒5.058], behavioral CVH (OR, 95% CI: 2.200, 1.603‒3.021), and biological CVH (OR, 95% CI: 2.567, 1.801‒3.657) at baseline. In the cross-sectional study, the OR values of BMI, blood pressure, blood glucose, and total cholesterol in the group with the highest grip strength were higher than those in the group with the lowest grip strength (*P* for trend < 0.05). However, the OR values of smoking in the group with the highest grip strength were lower than those in the group with the lowest grip strength (*P *< 0.05) (Supplementary Table S2).

**Table 4:** Association between relative handgrip strength and global CVH score, behavioral CVH score, biological CVH score in the cross-sectional study (*n* = 3,203).

		Handgrip strength/body mass			*P* for trend
		0.36 ≥ NGS > 0	0.46 ≥ NGS > 0.36	NGS > 0.46	
Global CVH Score	Crude Model	1.0 (Reference)	2.184 (1.650,2.890)	1.997 (1.505,2.651)	<0.001
	Model I	1.0 (Reference)	2.340 (1.754,3.122)	3.636 (2.604,5.076)	<0.001
	Model II	1.0 (Reference)	2.358 (1.763,3.153)	3.613 (2.580,5.058)	<0.001
Behavioral CVH Score	Crude Model	1.0 (Reference)	1.246 (0.970,1.601)	1.063 (0.822,1.375)	0.648
	Model I	1.0 (Reference)	1.614 (1.245,2.093)	2.264 (1.652,3.102)	<0.001
	Model II	1.0 (Reference)	1.590 (1.224,2.064)	2.200 (1.603,3.021)	<0.001
Biological CVH Score	Crude Model	1.0 (Reference)	2.082 (1.554,2.791)	2.282 (1.708,3.048)	<0.001
	Model I	1.0 (Reference)	1.849 (1.360,2.514)	2.634 (1.856,3.738)	<0.001
	Model II	1.0 (Reference)	1.841 (1.351,2.509)	2.567 (1.801,3.657)	<0.001

Logistic regression analysis assess the correlation between relative grip strength and Global CVH Score, Behavioral CVH Score, Biological CVH Score.

Global CVH Score consists of the following 7 indicators: smoking, physical activity, body mass index, diet, total cholesterol, blood pressure, and fasting plasma glucose.

Behavioral CVH Score consists of the following 4 indicators: smoking, physical activity, body mass index, diet.

Biological CVH Score consists of the following 3 indicators: total cholesterol, blood pressure, fasting plasma glucose. NGS, normalized handgrip strength; CVH, cardiovascular health.

**Crude Model:** Adjust for none; **Model I:** Adjust for age, sex**; Model II:** Adjust for age, sex, Education, Ethnicity, History of stroke, History of coronary heart disease.

### 3.2. Association between grip strength and ideal CVH in the follow-up study

Table 5 presents the adjusted relationships between grip strength and Ideal Global CVH Score in follow-up study. As summarized in Table 5, the maximum grip strength group had better global CVH (OR, 95% CI: 3.763, 1.805‒7.844), behavioral CVH (OR, 95% CI: 2.580, 1.333‒4.990), and biological CVH (OR, 95% CI: 2.164, 0.999‒4.686) in the follow-up study. In the follow-up study, there was no trend for smoking or blood pressure (Supplementary Table S3).

**Table 5:** Association between relative handgrip strength and global CVH score, behavioral CVH score, biological CVH score of Chinese rural adults in follow-up study (*n* = 761).

		Handgrip strength/body mass			*P* for trend
		0.36 ≥ NGS > 0	0.45 ≥ NGS > 0.36	NGS > 0.45	
Global CVH Score	Crude Model	1.0 (Reference)	2.054 (1.073,3.931)	2.643 (1.408,4.962)	0.003
	Model I	1.0 (Reference)	2.100 (1.082,4.076)	3.735 (1.802,7.744)	<0.001
	Model II	1.0 (Reference)	2.058 (1.058,4.005)	3.763 (1.805,7.844)	<0.001
Behavioral CVH Score	Crude Model	1.0 (Reference)	1.787 (1.061,3.011)	1.361 (0.791,2.344)	0.291
	Model I	1.0 (Reference)	2.301 (1.337,3.960)	2.555 (1.331,4.903)	0.003
	Model II	1.0 (Reference)	2.252 (1.303,3.891)	2.580 (1.333,4.990)	0.003
Biological CVH Score	Crude Model	1.0 (Reference)	2.134 (1.118,4.071)	2.143 (1.123,4.090)	0.026
	Model I	1.0 (Reference)	1.907 (0.976,3.726)	2.114 (0.985,4.534)	0.054
	Model II	1.0 (Reference)	1.898 (0.967,3.727)	2.164 (0.999,4.686)	0.049

Logistic regression analysis assess the correlation between relative grip strength and Global CVH Score, Behavioral CVH Score, Biological CVH Score.

Global CVH Score consists of the following 7 indicators: smoking, physical activity, body mass index, diet, total cholesterol, blood pressure, and fasting plasma glucose.

Behavioral CVH Score consists of the following 4 indicators: smoking, physical activity, body mass index, diet.

Biological CVH Score consists of the following 3 indicators: total cholesterol, blood pressure, fasting plasma glucose. NGS, normalized handgrip strength; CVH, cardiovascular health.

**Crude Model:** Adjust for none; **Model I:** Adjust for age, sex**; Model II:** Adjust for age, sex, Education, Ethnicity, History of stroke, History of coronary heart disease.

## 4. Discussion

Based on data from the cross-sectional study, our study showed that the ideal CVH rate was 12.4% in the rural area of China. The results suggest that after adjusting for confounding factors, high grip strength led to better ideal CVH, as compared with low grip strength. This association did not change in this cohort study. Among the CVH metrics, BMI, blood glucose, and total cholesterol showed a better trend with increased grip strength. Previous studies have suggested that poor rural family environment and low nutritional status of parents were negatively associated with midlife physical capability (30). Moreover, similar to some studies, we found that grip strength may be an under-recognized and controllable determinant of cardiometabolic risk factors in middle-aged and older adults in rural China (31).

Our study found that the proportion of ideal CVH was 12.4% in rural China, which was lower than that in developed countries, but higher than that in some developing countries (32–34). Some studies have shown that approximately 16% of participants have ideal CVH when 5–7 ideal indicators are identified (35). Other studies have shown that 7.8% of participants had ideal CVH metrics in ELSA-Brasil (33). Ideal CVH in rural China has become a serious public health concern. Consistent with previous reports, women were more often at the ideal level for smoking and blood pressure than were men; however, they had worse outcomes than did men in total cholesterol levels (36). In this study, no difference in diet between men and women was observed, which was related to the similar diet of rural residents. Our study also found that women had a lower ideal BMI than did men, which differed from the results of previous studies (36). Another reason may be the low educational level of local women. Studies in rural China have shown that education level is negatively correlated with general and abdominal obesity in women (37). At baseline, compared with other CVH studies in rural China, the ideal levels of BMI, total cholesterol, and fasting plasma glucose in this area were low and need to be improved.

Currently, little research on this topic exists, and previous studies have shown that relative grip strength is positively correlated with ideal CVH indicators in children and adolescents (24, 38). Another study on college students showed that the level of ideal CVH increases with increased grip strength (25). Ramírez-Vélez et al. provided evidence of an association between the components of ideal CVH and grip strength, which was partly consistent with the results of this study (24). However, few studies have examined the relationship between relative grip strength and the ideal CVH in rural China, where grip strength levels and ideal CVH differed from those in developed countries.

In the cross-sectional study, we found a link between glucose, total cholesterol, and muscular strength. This is consistent with a study showing that low-load resistance training is safe and beneficial in improving blood glucose and total cholesterol levels (19). In this study, we also found that as grip strength increased, the ideal level of blood pressure increased. This was consistent with studies of older adults in the community, which showed that isometric grip strength training resulted in a significant decrease of 9 mmHg in resting systolic blood pressure (21).In addition, in this cross-sectional study, BMI and muscle strength were correlated, which was consistent with the results of a previous study (39). To date, the effect of grip strength on ideal behavioral CVH has not been well established. In the cross-sectional study, neither physical activity nor diet were found to increase the ideal CVH level with the increase in grip strength, which is different from the results of previous studies. Previous cross-sectional and cohort studies have shown a positive relationship between grip strength and physical activity (40).

In our follow-up study, the correlations between grip strength and BMI, total cholesterol, and blood glucose were consistent with the results of the cross-sectional and previous studies. Insulin-stimulated glucose uptake occurs primarily in skeletal muscle, highlighting its importance in glucose control (41). The loss of skeletal muscle mass results in insulin resistance and reduced protein synthesis (42). Relative grip strength is positively correlated with ideal fasting glucose levels but negatively correlated with abdominal obesity and obesity (23, 39). Sarcopenia may be aggravated by obesity with excessive intramuscular fat deposition, probably because muscle loss and fat accumulation act synergistically (43). In addition, higher levels of C-reactive protein, interleukin6, and tumor necrosis factor*α* are associated with lower handgrip strength (44, 45). A study examining the effects of low-load resistance training on functional health and metabolic biomarkers in older women suggested that low-load resistance training is safe and beneficial in improving metabolic biomarkers, such as blood glucose and total cholesterol levels (19).

However, in our follow-up study, we found that smoking, physical activity, diet, and blood pressure did not improve as grip strength increased, which differed from the results of previous studies (24). The reason may be that in our study, local residents were mostly engaged in agricultural activities, and improvement in grip strength was partly related to occupation. This weakened the association between physical activity and grip strength. As for diet, there is less research on the relationship between grip strength and a healthy diet in China; hence, it is difficult to compare results. A study from outside China showed a correlation between adherence to the Mediterranean- Dietary Approaches to Stop Hypertension Intervention for Neurodegenerative Delay (MIND) diet pattern and better muscle strength (46). However, further research is needed to confirm these findings. Smoking and blood pressure did not rise as grip strength increased in the follow-up study, as compared to the cross-sectional study. In terms of blood pressure, a randomized controlled trial found that isometric handgrip exercise can lead to reductions in resting systolic blood pressure in older adults (21). In individuals with hypertension, isometric handgrip training can also tend to lower central systolic blood pressure (47). Our study did not find a significant association between a more ideal blood pressure status and an increase in grip strength in the cohort, which may be attributed to the limitation of a single grip strength test that may not accurately reflect long-term strength training.

Previous studies have indicated that grip strength is a better predictor of cardiovascular mortality than systolic blood pressure (48). Our study found a positive association between grip strength and ideal CVH from the cross-sectional and cohort study. In cross-sectional and follow-up studies, ideal BMI, total cholesterol, and blood glucose levels rose with increasing grip strength. To ensure the best cardiovascular health for local residents, grip strength levels need to be improved. Among the seven ideal cardiovascular health metrics, the ideal ratio of diet and blood pressure was low. To improve their cardiovascular health, locals should be urged to adopt good eating habits and intensify blood pressure monitoring. More research is needed to refine the mechanisms underlying the association between grip strength and ideal CVH components. In conclusion, our study is particularly important from a public health perspective, given the importance of determining the relationship between grip strength and ideal CVH status and the direction of improvement needed in rural areas for future CVH in these regions. We conducted a cohort study of grip strength and ideal CVH in middle-aged and older adults. We simultaneously also conducted a preliminary analysis and explored the components of ideal CVH and grip strength.

The strength of our study is that it described ideal CVH in rural areas of China, where grip strength levels and ideal CVH differ from those in urban and other developed countries. We further explored the relationship between relative grip strength and ideal cardiovascular score and its components in rural areas in a follow-up study. In the light of our results, some limitations of this study should be considered. First, we only surveyed rural areas in the Liaoning province, where a higher proportion of the Mongolian population is located; these individuals are less educated and have less access to healthcare, limiting the generalizability of our study findings. Further verification is required for other Chinese provinces. Second, owing to the COVID-19 pandemic and the lack of contact information of some participants, the follow-up rate was not very high, which needs to be improved in future studies.

In conclusion, our study found that the ideal CVH status in rural areas of the Liaoning province was low. The ideal CVH of men was 6.8%, whereas that of women was 15.7%, which was more than twice that of men. We report that, compared with high grip strength, low grip strength resulted in lower ideal CVH levels. Grip strength is thus a rough predictor of ideal CVH. More attention should be paid to ideal CVH in rural China, and particularly to the men in these rural areas. To improve ideal CVH in rural areas, it is important to strengthen the control of smoking in rural men, while it is essential to encourage women to control their BMI and blood glucose levels. Strengthening grip strength levels in both men and women in rural areas can improve local ideal CVH.

## Data Availability

The raw data supporting the conclusions of this article will be made available by the authors, without undue reservation.
