# Supplementary material for: Prevalence of ideal cardiovascular health and its relationship with relative handgrip strength in rural northeast China
Source: Front Cardiovasc Med. 2023 Jun 2;10:1124757. doi: 10.3389/fcvm.2023.1124757 (PMC10275610; doi:10.3389/fcvm.2023.1124757)
Supplement: Supplementary file 1 [file Table1.docx]

**Supplementary Table1** Total cardiovascular health score and definitions of cardiovascular health

| Criteria | Ideal (5-7 Metrics) | Intermediate (3-4 Metrics) | Poor (0-2 Metrics) |
| --- | --- | --- | --- |
| Smoking | Never or quit >12 months | Former ≤12 months | Current |
| Physical activity | ≥150 minutes moderate/vigorous  activity per wk | 1 to 149 minutes moderate/vigorous  activity per wk | No physical activity |
| Body mass index | <25 kg/m^2^ | 25 to 29.9 kg/m^2^ | ≥30 kg/m^2^ |
| Diet | 4 to 5 dietary components | 2 to 3 dietary components | 0 to 1 dietary components |
| Blood pressure | SBP <120 and DBP <80 mm Hg | SBP 120 to 139 or DBP 80 to 89, or treated to SBP <140/DBP <90 mm Hg | SBP ≥140 or DBP≥90 mm Hg |
| Fasting plasma glucose | <100 mg/dL | 100 to 125 or treated to <100 mg/dL | ≥126 mg/dL |
| Total cholesterol | <200 mg/dL | 200 to 239 or treated to <200 mg/dL | ≥240 mg/dL |
| No. of ideal cardiovascular health indicators | 5-7 Metrics | 3-4 Metrics | 0-2 Metrics |

**Supplementary Table2.** Association between Relative handgrip strength and Ideal Global CVH components of Chinese rural adults in the cross-sectional study (n =3203).

|  | Handgrip strength/body mass | | | *P* for trend | | |
| --- | --- | --- | --- | --- | --- | --- |
|  | 0.36≥NGS＞0 | 0.46≥NGS＞0.36 | NGS＞0.46 | |  |  |
| Ideal Smoking | 1.0 (Reference) | 0.867 (0.684,1.100) | 0.457 (0.352,0.594) | | ＜0.001 |  |
| Ideal Physical activity | 1.0 (Reference) | 0.916(0.731,1.149) | 0.979(0.742,1.291) | | 0.812 |  |
| Ideal Body mass index | 1.0 (Reference) | 3.094(2.551,3.752) | 12.251(9.427,15.921) | | ＜0.001 |  |
| Ideal Diet | 1.0 (Reference) | 1.106(0.878,1.394) | 1.272(0.965,1.677) | | 0.089 |  |
| Ideal Blood pressure | 1.0 (Reference) | 1.332(1.077,1.647) | 1.830(1.411,2.375) | | ＜0.001 |  |
| Ideal Fasting plasma glucose | 1.0 (Reference) | 1.539(1.284,1.844) | 1.833(1.467,2.289) | | ＜0.001 |  |
| Ideal Total cholesterol | 1.0 (Reference) | 1.194(0.997,1.429) | 1.245(1.001,1.550) | | 0.042 |  |

Global CVH consists of the following 7 indicators: smoking, physical activity, body mass index, diet, total cholesterol, blood pressure, and fasting plasma glucose.

NGS, normalized handgrip strength; CVH, cardiovascular health.

Adjust for age; sex; Education; Ethnicity; History of stroke; History of coronary heart disease.

**Supplementary Table3.** Association between Relative handgrip strength and Ideal Global CVH components of Chinese rural adults in follow-up study (n =761).

|  | Handgrip strength/body mass | | | *P* for trend | | |
| --- | --- | --- | --- | --- | --- | --- |
|  | 0.37≥NGS＞0 | 0.46≥NGS＞0.37 | NGS＞0.46 | |  |  |
| Ideal Smoking | 1.0 (Reference) | 0.810(0.486,1.350) | 0.792(0.447,1.403) | | 0.446 |  |
| Ideal Physical activity | 1.0 (Reference) | 1.142(0.708,1.844) | 1.154(0.639,2.081) | | 0.609 |  |
| Ideal Body mass index | 1.0 (Reference) | 4.911(3.240,7.444) | 17.728(10.197,30.821) | | ＜0.001 |  |
| Ideal Diet | 1.0 (Reference) | 1.319(0.751,2.317) | 1.154(0.578,2.303) | | 0.637 |  |
| Ideal Blood pressure | 1.0 (Reference) | 1.408(0.849,2.334) | 1.279(0.697,2.346) | | 0.394 |  |
| Ideal Fasting plasma glucose | 1.0 (Reference) | 1.467(1.015,2.121) | 1.899(1.214,2.972) | | 0.004 |  |
| Ideal Total cholesterol | 1.0 (Reference) | 1.169(0.800,1.708) | 1.719(1.074,2.752) | | 0.028 |  |

Global CVH consists of the following 7 indicators: smoking, physical activity, body mass index, diet, total cholesterol, blood pressure, and fasting plasma glucose.

NGS, normalized handgrip strength; CVH, cardiovascular health.

Adjust for age; sex; Education; Ethnicity; History of stroke; History of coronary heart disease.


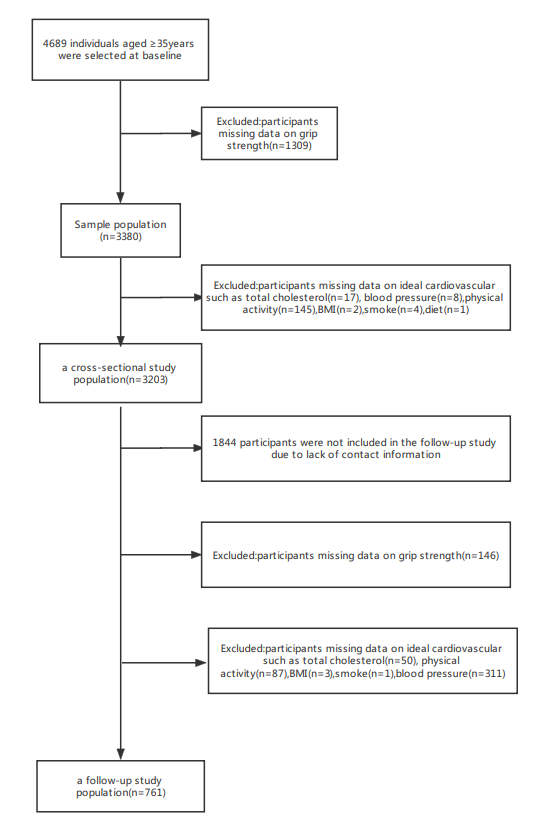


**Supplementary Figure 1** The inclusion and exclusion criteria for the study population.
